# ^111^In-anti-F4/80-A3-1 antibody: a novel tracer to image macrophages

**DOI:** 10.1007/s00259-015-3084-8

**Published:** 2015-05-27

**Authors:** Samantha Y. A. Terry, Otto C. Boerman, Danny Gerrits, Gerben M. Franssen, Josbert M. Metselaar, Steffi Lehmann, Wim J. G. Oyen, Christian A. Gerdes, Keelara Abiraj

**Affiliations:** Department of Radiology and Nuclear Medicine, Radboud University Medical Center, Nijmegen, The Netherlands; Department of Imaging Chemistry and Biology, King’s College London, St Thomas’ Hospital, London, UK; Targeted Therapeutics, MIRA Institute for Biomedical Technology and Technical Medicine, University of Twente, Enschede, The Netherlands; Roche Pharmaceutical Research and Early Development (pRED), Roche Innovation Center Zurich, Zurich, Switzerland; Roche Pharmaceutical Research and Early Development (pRED), Roche Innovation Center Basel, Basel, Switzerland

**Keywords:** Macrophages, Tumour-associated macrophages, F4/80, Imaging, ^111^In

## Abstract

**Purpose:**

Here, the expression of F4/80 on the cell surface of murine macrophages was exploited to develop a novel imaging tracer that could visualize macrophages in vivo.

**Methods:**

The immunoreactive fraction and IC_50_ of anti-F4/80-A3-1, conjugated with diethylenetriaminepentaacetic acid (DTPA) and radiolabelled with ^111^In, were determined in vitro using murine bone marrow-derived macrophages. In vivo biodistribution studies were performed with ^111^In-anti-F4/80-A3-1 and isotype-matched control antibody ^111^In-rat IgG2b at 24 and 72 h post-injection (p.i.) in SCID/Beige mice bearing orthotopic MDA-MB-231 xenografts. In some studies mice were also treated with liposomal clodronate. Macrophage content in tissues was determined immunohistochemically. Micro-single photon emission computed tomography (SPECT)/CT images were also acquired.

**Results:**

In vitro binding assays showed that ^111^In-anti-F4/80-A3-1 specifically binds F4/80 receptor-positive macrophages. The immunoreactivity of anti-F4/80-A3-1 was 75 % and IC_50_ was 0.58 nM. In vivo, injection of 10 or 100 μg ^111^In-anti-F4/80-A3-1 resulted in splenic uptake of 78 %ID/g and 31 %ID/g, respectively, and tumour uptake of 1.38 %ID/g and 4.08 %ID/g, respectively (72 h p.i.). Liposomal clodronate treatment reduced splenic uptake of 10 μg ^111^In-anti-F4/80-A3-1 from 248 %ID/g to 114 %ID/g and reduced ^111^In-anti-F4/80-A3-1 uptake in the liver and femur (24 h p.i.). Tracer retention in the blood and tumour uptake increased (24 h p.i.). Tumour uptake of ^111^In-anti-F4/80-A3-1 was visualized by microSPECT/CT. Macrophage density in the spleen and liver decreased in mice treated with liposomal clodronate. Uptake of ^111^In-rat IgG2b was lower in the spleen, liver and femur when compared to ^111^In-anti-F4/80-A3-1.

**Conclusion:**

Radiolabelled anti-F4/80-A3-1 antibodies specifically localize in tissues infiltrated by macrophages in mice and can be used to visualize tumours. The liver and spleen act as antigen sink organs for macrophage-specific tracers.

**Electronic supplementary material:**

The online version of this article (doi:10.1007/s00259-015-3084-8) contains supplementary material, which is available to authorized users.

## Introduction

Macrophages play an important role in the phagocytosis of microbes and in the regulation of developmental processes and pathophysiological responses, including inflammation and tumour growth [1]. Monocytes, once resident in tissues, acquire tissue-specific characteristics and express cell surface proteins in response to signals within the microenvironment, which determine their local function. During acute inflammation, M1 macrophages become activated through a classical pathway and are able to kill and phagocytose tumour cells. M2 macrophages, activated alternatively, are involved in key processes of tumour progression, including angiogenesis, metastasis and immunosuppression [2].

The F4/80 receptor is generally considered a murine pan-macrophage marker [3] and is expressed on tumour-associated macrophages (TAMs). The production of anti-F4/80-A3-1 antibodies was first reported in 1981 [3]. The expression of the F4/80 receptor on macrophages plays a role in peripheral tolerance [4] and T cell-mediated immune response [5]. Expression can occur on mature bone marrow stromal macrophages, Kupffer cells [6], eosinophils after parasitic infection [7] and in alveoli, albeit at low levels [8]. Although M1 and M2 macrophage phenotypes are not stable as they can change in response to environmental signals [9], TAMs mostly polarize towards the M2 phenotype [10]. Consequently, the density of TAMs is associated with poor prognosis [11–15]. Therapies targeting the interaction between TAMs and tumour cells have therefore gained much interest and tend to focus either on polarizing TAMs towards a tumouricidal phenotype using agonists, inhibitors and other agents or on decreasing the number of TAMs in tumour tissue [16–20].

The multiple therapies targeting TAMs could offer new routes in cancer therapy. Being able to image TAMs could aid in the development of novel macrophage-targeted drugs and could be applied to monitor the kinetics of macrophage infiltration in response to antibody-based immunotherapies engaging innate immune cells, such as therapy with anti-epidermal growth factor receptor (EGFR) antibodies [16]. In addition, TAM-specific radiotracers might ultimately allow the monitoring of prognosis of patients or aid the selection of patients that are more likely to respond to these therapies.

Macrophages and other phagocytic cells have previously been imaged through passive uptake of radiolabelled PEGylated, mannosylated liposomes, and dextran nanoparticles [21–23], the dual magneto-optical imaging probe PG-Gd-NIR813 [24], and iron oxide or functionally derivatized nanoparticles [25, 26]. More targeted methods of non-invasively imaging TAMs include ^99m^Tc-labelled anti-mannose receptor nanobodies [27] and radiolabelled folic acid [28].

In this article, we describe the radiolabelling and in vitro and in vivo characterization of a novel anti-F4/80-A3-1 antibody-based radiotracer for specific imaging of macrophages.

## Materials and methods

### Synthesis of ITC-DTPA-conjugated antibodies

Rat anti-mouse anti-F4/80-A3-1 antibody (AbD Serotec, Kidlington, UK; monoclonal; clone Cl:A3-1) and rat IgG2b isotype control (R&D Systems, Abingdon, UK) were dialysed against 1:1 phosphate-buffered saline (PBS):water to remove sodium azide. Antibodies were conjugated under strict metal-free conditions with isothiocyanatobenzyl-diethylenetriaminepentaacetic acid (ITC-DTPA, Macrocyclics, Dallas, TX, USA) in 0.1 M NaHCO_3_, pH 9.5, using a 10-fold molar excess of ITC-DTPA for 1 h at room temperature (RT). Unbound ITC-DTPA was removed by dialysis against 0.1 M 2-(*N*-morpholino)ethanesulfonic acid (MES), pH 5.5.

### Radiolabelling

^111^InCl_3_ (Mallinckrodt, Petten, The Netherlands) was added to ITC-DTPA-anti-F4/80-A3-1 or -rat IgG2b for 1 h at RT in 0.1 M MES buffer pH 5.4. For biodistribution studies, approximately 80 MBq was added to 4 μg ITC-DTPA-anti-F4/80-A3-1 or -rat IgG2b. For single photon emission computed tomography (SPECT) studies, 43 MBq was added to 20 μg ITC-DTPA-anti-F4/80-A3-1 or -rat IgG2b. Free ^111^In was complexed by adding ethylenediaminetetraacetic acid (EDTA; final concentration of 5 mM). Labelling efficiency and radiochemical purity were determined using instant thin-layer chromatography and fast protein liquid chromatography (see Supplementary figures for details). To obtain a radiochemical purity ≥95 %, ^111^In-labelled antibodies were purified by gel filtration on a PD10 column (GE Healthcare Life Sciences) eluted with PBS containing 0.5 % bovine serum albumin (BSA).

### Isolation and ex vivo differentiation of macrophages

Intact femurs were removed from SCID/Beige mice, euthanized by CO_2_/O_2_, and placed into PBS on ice. Under sterile conditions, femurs were submerged in 70 % ethanol, PBS and Dulbecco’s modified Eagle’s medium (DMEM) and Ham’s F12 (DMEM-F12). Femurs were flushed with DMEM-F12 and harvested bone marrow cells (10 × 10^6^ cells per femur) were pelleted at 300 *g* for 5 min at 4 °C, filtered through a 100-μm nylon mesh (BD Biosciences) and plated at 10 × 10^6^ cells per 100 × 20 mm dish in DMEM-F12 with 10 % fetal calf serum (FCS; Invitrogen; Life Technologies), 1 % glutamine, 1 % penicillin/streptomycin (Invitrogen) and 100 μg/ml recombinant mouse M-CSF (R&D Systems) (full DMEM-F12) at 37 °C in a humidified 5 % CO_2_ atmosphere for 7 days in total, before being harvested by heat shock from 37 to 4 °C. Animal experiments were approved by the local Animal Welfare Committee in accordance with Dutch legislation and carried out in accordance with their guidelines.

### Cell culture

MDA-MB-231 human breast cancer cells, negative for F4/80, were cultured in RPMI-1640 supplemented with 10 % (v/v) FCS and 1 % glutamine (Invitrogen). Cells were maintained at 37 °C in a humidified 5 % CO_2_ atmosphere and routinely passaged using a 0.25 % trypsin/EDTA solution (Invitrogen).

### Flow cytometry

Macrophages (0.5 × 10^6^) were stained with anti-mouse CD11b-FITC and anti-mouse F4/80-PE antibodies (Biolegend) at 4 °C for 30 min in PBS with 0.5 % BSA. Cells (10,000) were analysed with a FACSCalibur (BD Biosciences) using forward/side scatter characteristics and analysed using CellQuest software (BD Biosciences). Samples stained with each fluorophore separately were used to alter voltage and amplitude gain settings to allow for compensation.

### In vitro binding assays

Immunoreactive fractions of ^111^In-anti-F4/80-A3-1 and ^111^In-rat IgG2b were determined as described by Lindmo et al. [29]. A serial dilution of cells (1 ml) was prepared in DMEM-F12 supplemented with 0.5 % BSA; 2 kBq of radiolabelled tracer (1 ng) was added. Non-specific binding was determined by incubation in the presence of a blocking dose of unlabelled antibody (10 μg). After 30 min at 37 °C, cells were centrifuged, washed and the supernatant collected. Pellets were lysed in 0.1 M NaOH. The activity in the supernatant (unbound) and pellets (bound) was measured in a gamma counter.

The concentration required to inhibit binding of ^111^In-anti-F4/80-A3-1 by 50 % (IC_50_) was determined using 5 × 10^6^ macrophages in DMEM-F12 supplemented with 0.5 % BSA incubated with increasing concentrations of ITC-DTPA-anti-F4/80-A3-1 (50 pM to 70 nM) and 2 kBq of radiolabelled tracer (1 ng). After 30 min incubation on ice and washing, cell-bound activity was measured in a gamma counter. Data were analysed using GraphPad Prism (version 5.03).

### Production of liposomes

Clodronate liposomes were prepared by injecting 1 ml of a lipid solution of 1 mmol/ml in ethanol [containing dipalmitoyl phosphatidylcholine (DPPC), dipalmitoyl phosphatidylglycerol (DPPG) (both from Lipoid GmbH, Ludwigshafen, Germany) and cholesterol (Sigma-Aldrich) in a molar percentage of 62, 5 and 33 % of total lipid, respectively] in 9 ml of an aqueous solution of 100 mg/ml clodronate disodium salt (Sigma-Aldrich). Subsequently, the 10 ml crude liposome dispersion was sized by multiple extrusion at 60 °C using a medium pressure extruder (Avestin, Mannheim, Germany) equipped with two stacked polycarbonate membrane filters, one with a pore size of 200 nm on top of one with 100 nm pores. Alcohol and free clodronate (not incorporated in liposomes) were removed by repeated cycles of ultrafiltration and replacement of the filtrate with PBS. The resulting formulation consisted of liposomes of approximately 125 nm in diameter as measured by dynamic light scattering, with a polydispersity index of 0.05 and a zeta potential of approximately −30 mV. Content determination was done by extraction using the organic phase for lipid determination (HPLC followed by evaporative light scattering detection) and the aqueous phase to assess the clodronate content (UV spectrophotometry at 238 nm after formation of clodronate complex with CuSO_4_ solution). The liposomes contained approximately 2 mg clodronate/ml and 70 μmol total lipid/ml. Empty liposomes were prepared in the same manner using PBS instead of the aqueous clodronate solution.

### Biodistribution studies

In biodistribution studies, female SCID/Beige mice were inoculated in the mammary fad pad with 50 μl RPMI-1640 medium containing 2 × 10^6^ MDA-MB-231 cells supplemented with BD Matrigel Basement Membrane Matrix (BD Biosciences, Franklin Lakes, NJ, USA). When tumours reached 150–400 mm^3^ in volume, mice were injected intravenously (i.v.) with purified 10 μg (1 MBq) or 100 μg ^111^In-anti-F4/80-A3-1 (1.6 MBq) or ^111^In-rat IgG2b (1.5 MBq) in 200 μl PBS, 0.5 % BSA. Mice were euthanized by CO_2_/O_2_ asphyxiation at 24 or 72 h post-injection (p.i.). For biodistribution studies involving liposomes, 300 μl of the liposome mix was injected i.v. per mouse, twice in 4 days (5 days and 24 h, i.e. just prior to injection of the tracer, before euthanasia). Blood, tumour and the major organs and tissues were dissected, weighed and counted in a gamma counter. Blood samples were also taken at 1 h p.i. of 10 μg ^111^In-F4/80 or ^111^In-rat IgG2b in mice treated with or without clodronate or empty liposomes. Tumours, spleens and livers were flash frozen for further immunohistochemical analysis. The percentage injected dose per gram (%ID/g) of each sample was calculated.

### Immunohistochemistry

Tissues were snap-frozen and sections were fixed in acetone, blocked for 30 min with 5 % normal goat serum and incubated with rat anti-mouse anti-CD68 antibody (AbD Serotec) followed by a secondary biotinylated goat anti-rat antibody. Sections were then exposed to an avidin-biotin-enzyme complex (Vector Laboratories, Burlingame, CA, USA) and were incubated with diaminobenzidine for development followed by a haematoxylin counterstain. The number of CD68-positive cells per 1.5 mm × 1.5 mm square (as highlighted in ×10 image) was calculated for the spleen, liver and tumour sections averaged from six to eight squares/areas of tissue from one sample.

### MicroSPECT imaging

In microSPECT imaging studies, mice with orthotopic MDA-MB-231 tumour xenografts were injected i.v. with 100 μg purified ^111^In-F4/80 (14 MBq). Mice were euthanized at 24 h p.i. and scanned using the U-SPECT-II/CT (4 frames of 15 min; spatial resolution 160 μm, 65 kV, 615 μA) (MILabs) [30]. A CT scan was also taken for anatomic reference. Mice were euthanized prior to imaging, rather than imaged under anaesthesia, to allow a proper comparison of the SPECT/CT imaging data with the biodistribution data. SPECT scans, four frames combined, were reconstructed using an ordered subset expectation maximization algorithm, with a voxel size of 0.4 mm (MILabs).

### Statistical analysis

In in vitro and biodistribution studies, all values are given as mean ± standard deviation. Statistical analysis in biodistribution studies and tabular data (Fig. 3 and Table 1) was performed using a two-way analysis of variance (ANOVA) with Tukey’s multiple comparisons test with GraphPad Prism Software (version 5.03). Other data were analysed by a one-sample *t* test. Statistical significance was set at *p ≤ 0.05, **p ≤ 0.01, ***p ≤ 0.001 and ****p ≤ 0.0001.

**Table 1:** Uptake (%ID/g) of 10 and 100 μg ^111^In-anti-F4/80-A3-1 at 24 and 72 h p.i. in tissues and organs of SCID/Beige mice with orthotopic MDA-MB-231 tumours

	10 μg at 24 h	10 μg at 72 h	100 μg at 72 h
Blood	1.27 ± 0.61	0.39 ± 0.31	0.42 ± 0.16
Muscle	0.20 ± 0.06	0.10 ± 0.01	0.25 ± 0.06
Tumour	2.01 ± 0.20	1.38 ± 0.34	4.08 ± 0.27
Spleen	247.88 ± 61.94	78.23 ± 8.22	30.9 ± 5.51
Pancreas	0.78 ± 0.28	0.66 ± 0.32	1.17 ± 0.16
Kidney	17.35 ± 1.80	8.15 ± 0.95	11.10 ± 0.97
Stomach	0.73 ± 0.12	0.71 ± 0.28	1.23 ± 0.09
Duodenum	8.93 ± 3.88	4.06 ± 1.01	4.92 ± 0.49
Liver	28.43 ± 2.96	11.18 ± 2.88	9.04 ± 0.36
Tumour:blood	1.8 ± 0.6	4.6 ± 2.3	11.2 ± 4.8

Data are presented as average ± standard deviation (10 μg 24 h spleen, 100 μg 72 h *n* = 4; rest *n* = 5)

## Results

### Synthesis and radiolabelling of antibodies

^111^In-anti-F4/80-A3-1 and ^111^In-rat IgG2b were prepared with a maximum specific activity of 12 and 2 MBq/μg, respectively. After purification, a radiochemical purity of ≥95 % was achieved for both tracers (data not shown). A sodium dodecyl sulphate polyacrylamide gel electrophoresis (SDS-PAGE) gel was run in the evaluation of the antibody integrity, after conjugation, and it showed a band at both 140 and 50 kDa, suggesting the presence of a F(ab) fragment in the preparation.

### Flow cytometry

Bone marrow cells isolated from murine femoral bone marrow were differentiated to CD11b^+^/F4/80^+^ macrophages. MDA-MB-231 human breast cancer cells were CD11b^−^/F4/80^−^ (data not shown).

### In vitro binding assays

**Fig. 1:**
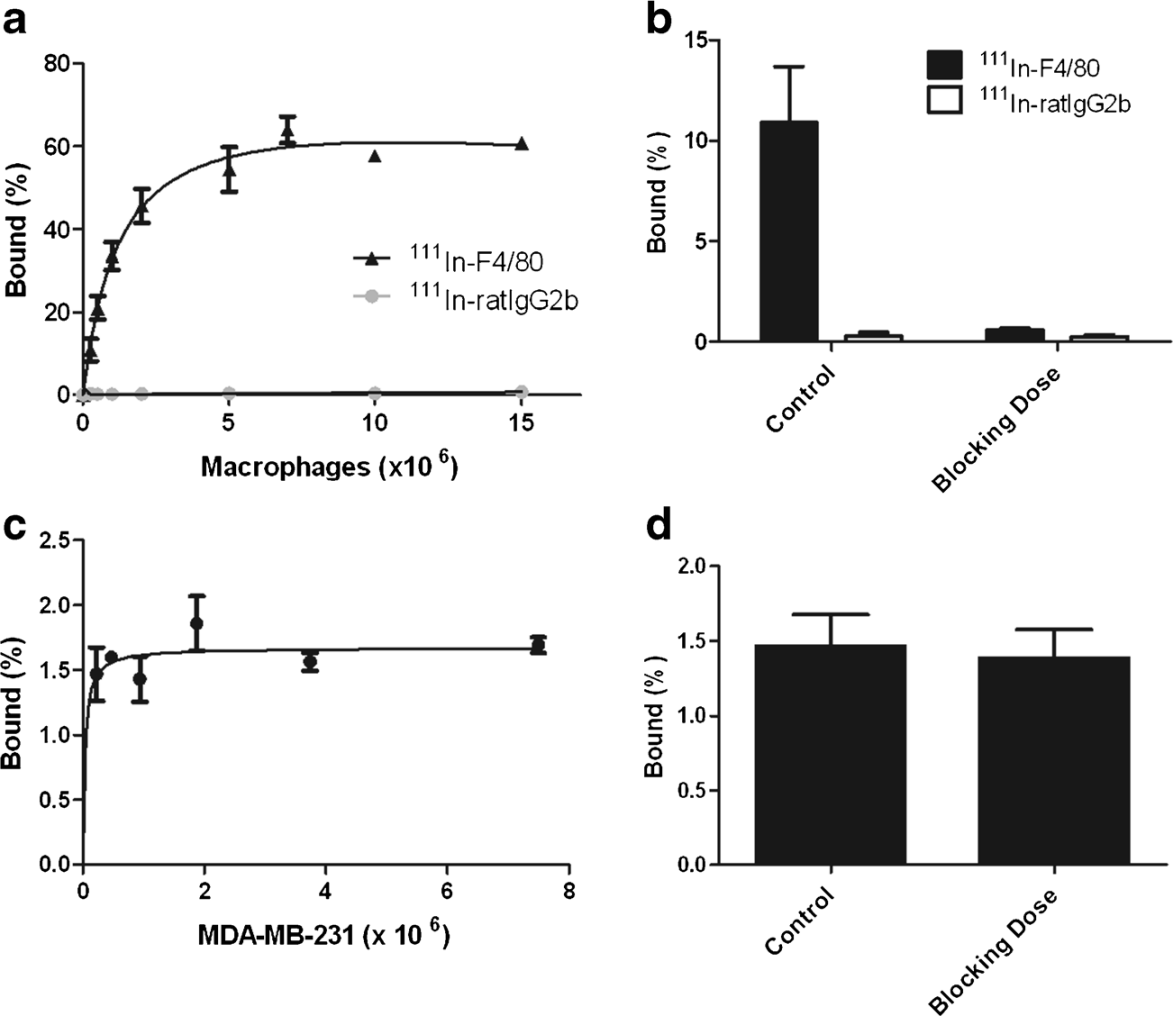
In vitro binding of ^111^In-anti-F4/80-A3-1 (**a**–**d**) or isotype control ^111^In-rat IgG2b (**a**, **b**) to macrophages (**a**, **b**) or MDA-MB-231 cells (**c**, **d**). Cells were also incubated in the presence of a blocking dose of antibody (**b**, **d**) to ascertain target-mediated binding of each tracer. Data are presented as mean ± standard deviation (^111^In-anti-F4/80-A3-1: *n* = 3/study; ^111^In-rat IgG2b: *n* = 2/study; experiments were carried out twice)

**Fig. 2:**
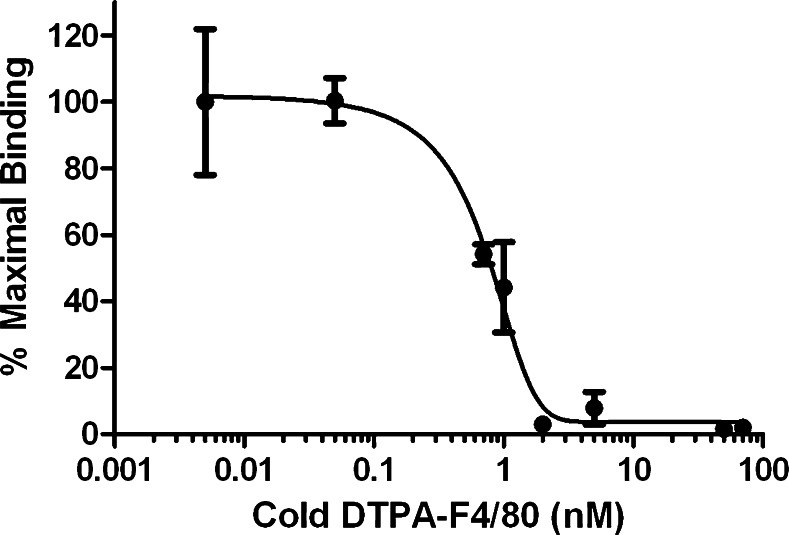
Competitive binding curve to determine the IC_50_ of ITC-DTPA-anti-F4/80-A3-1 for macrophages. ^111^In-anti-F4/80-A3-1 was used as a radioactive tracer. Data are presented as mean ± standard deviation (*n* = 2/study; experiments were carried out twice)

The immunoreactive fraction of ^111^In-anti-F4/80-A3-1 for F4/80 receptors was 75 % (Fig. 1a and Supplementary Figure 1). ^111^In-anti-F4/80-A3-1 did not bind MDA-MB-231 cells (Fig. 1c, d), nor did ^111^In-rat IgG2b bind F4/80-positive macrophages (Fig. 1a, b; *p* < 0.01). The addition of a blocking dose of ITC-DTPA-anti-F4/80-A3-1 decreased binding of ^111^In-anti-F4/80-A3-1 to macrophages from 10.9 ± 2.8 to 0.6 ± 0.1 % (Fig. 1b), indicating specific binding of ^111^In-anti-F4/80-A3-1 to macrophages. The IC_50_ was 0.58 nM (Fig. 2).

### Biodistribution studies

The results of the biodistribution studies of 10 and 100 μg ^111^In-anti-F4/80-A3-1 at 24 and 72 h p.i. are summarized in Table 1. ^111^In-anti-F4/80-A3-1 (10 μg) in the circulation decreased from 1.27 %ID/g at 24 h p.i. to 0.39 %ID/g at 72 h p.i. Uptake of ^111^In-anti-F4/80-A3-1 in the tumour, liver and spleen (*p* ≤ 0.001) also decreased; however, tracer uptake in all three tissues increased relative to blood. At 72 h p.i., the tumour to blood uptake ratio of ^111^In-anti-F4/80-A3-1 (10 μg) was 4.6 compared to 1.8 at 24 h p.i. The spleen to blood and liver to blood ratios also increased from 195 to 201 and from 22 to 29, at 24 and 72 h p.i., respectively.

Injection of 100 μg ^111^In-anti-F4/80-A3-1 resulted in a lower splenic uptake than injection of 10 μg ^111^In-anti-F4/80-A3-1: 30.94 %ID/g versus 78.23 %ID/g at 72 h p.i. (Table 1; *p* ≤ 0.0001). Uptake of ^111^In-anti-F4/80-A3-1 in the MDA-MB-231 tumour was higher at the higher dose: 4.08 %ID/g versus 1.38 %ID/g.

**Fig. 3:**
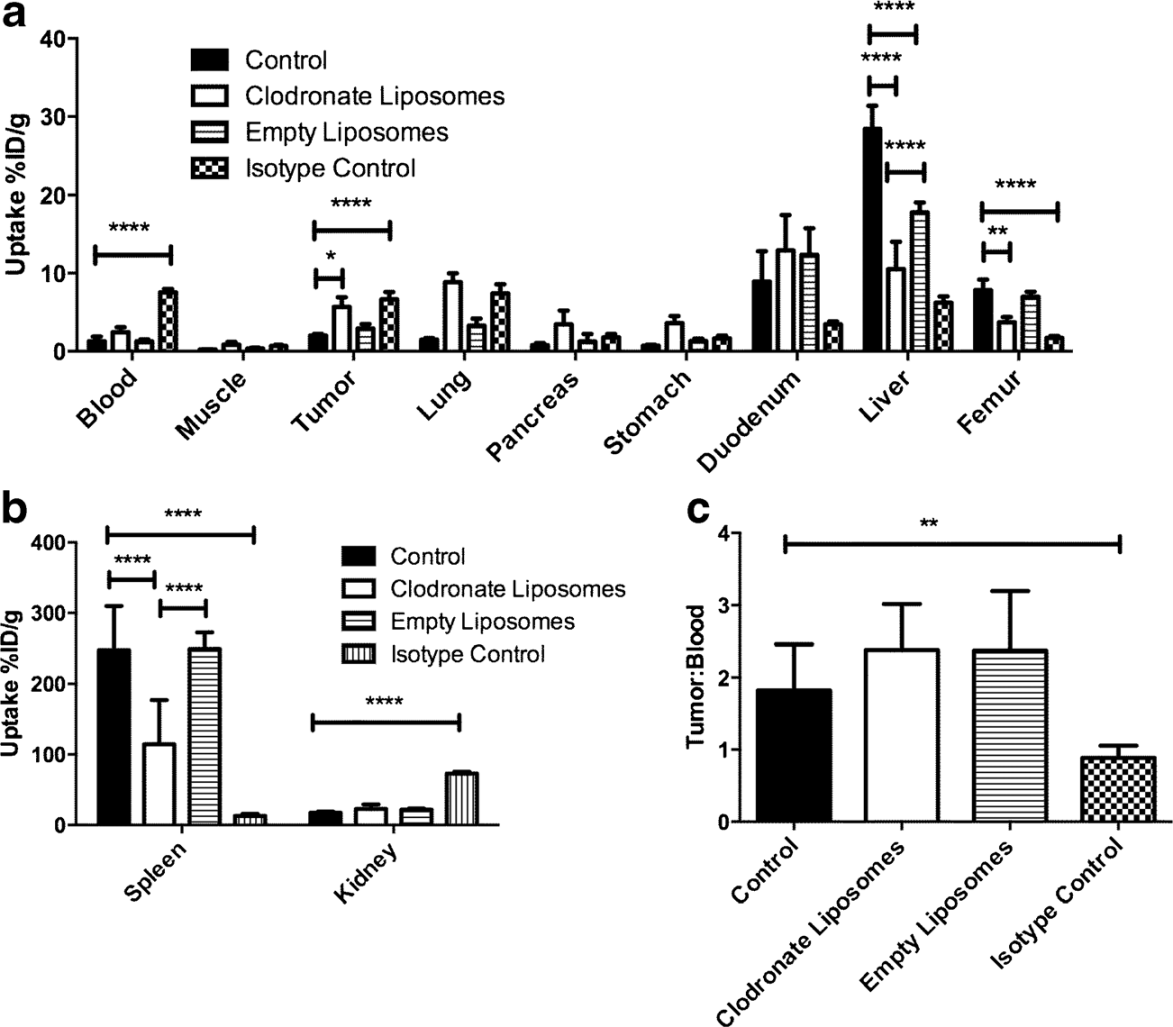
Biodistribution of 10 μg ^111^In-anti-F4/80-A3-1 or isotype control ^111^In-rat IgG2b at 24 h p.i. in tissues and organs (**a**–**b**) of mice treated with clodronate liposomes or empty liposomes (^111^In-anti-F4/80-A3-1). Isotype control =^111^In-rat IgG2b. Tumour to blood ratios of 10 μg ^111^In-anti-F4/80-A3-1 or ^111^In-rat IgG2b at 24 h p.i. in mice treated with clodronate liposomes or empty liposomes (^111^In-anti-F4/80-A3-1) or injected with isotype control (^111^In-rat IgG2b) were also calculated (**c**). All studies were performed using SCID/Beige mice with orthotopic MDA-MB-231 tumour xenografts. Data are mean ± standard deviation (spleen: *n* = 4; rest: *n* = 5)

Splenic uptake of ^111^In-anti-F4/80-A3-1 at 24 h p.i. was markedly reduced after mice were treated with clodronate liposomes: 114.35 %ID/g compared to 247.88 %ID/g (*p* ≤ 0.0001), whereas uptake was unaffected in mice treated with empty liposomes (*p* > 0.05; Fig. 3b). Splenic uptake of the isotype-matched control antibody ^111^In-rat IgG2b was markedly lower (12.70 %ID/g) than that of ^111^In-anti-F4/80-A3-1 (*p* ≤ 0.0001).

Clodronate liposomal treatment resulted in decreased uptake of ^111^In-anti-F4/80-A3-1 in both the liver (*p* ≤ 0.0001) and femur (*p* ≤ 0.01) (10.51 and 3.73 %ID/g, respectively) compared to the control group (28.43 and 7.81 %ID/g, respectively; Fig. 3a) at 24 h p.i. Most importantly, uptake of ^111^In-anti-F4/80-A3-1 in the tumour increased after clodronate treatment (5.67 %ID/g compared to 2.01 %ID/g; *p* ≤ 0.05). Figure 3c shows that tumour to blood ratios of ^111^In-anti-F4/80-A3-1 did not significantly differ in animals treated with or without clodronate liposomes (*p* > 0.05). Tumour uptake of ^111^In-rat IgG2b was significantly higher than ^111^In-anti-F4/80-A3-1 (Fig. 3a; *p* ≤ 0.0001), yet higher circulation levels of ^111^In-rat IgG2b meant significantly lower tumour to blood values for ^111^In-rat IgG2b than for ^111^In-anti-F4/80-A3-1. Blood levels of ^111^In-anti-F4/80-A3-1 (10 μg) at 1 h p.i. were clearly higher after clodronate treatment (50.43 %ID/g versus 6.18 %ID/g in control; Table 2).

**Table 2:** Retention of 10 μg ^111^In-anti-F4/80-A3-1 or control isotype ^111^In-rat IgG2b at 1 h p.i. in blood in tumour-bearing mice treated with vehicle, clodronate liposomes or empty liposomes

Control	Clodronate liposomes	Empty liposomes	Isotype control
6.18 ± 0.76	50.43 ± 11.07	21.77 ± 3.59	32.37 ± 0.97

Data are presented as mean ± standard deviation (*n* = 2)

### Immunohistochemistry

**Fig. 4:**
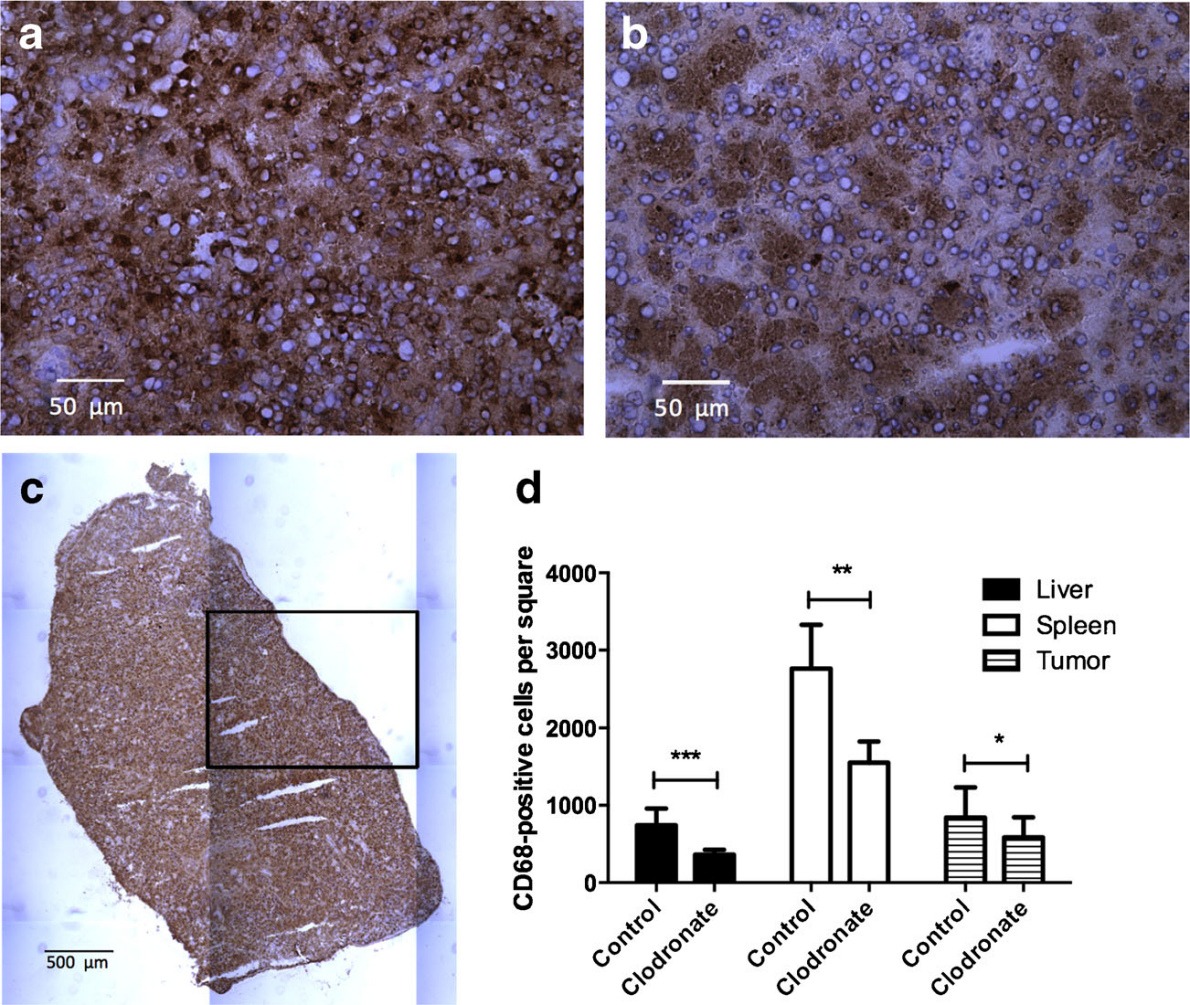
Immunohistochemical staining for macrophages with anti-CD68 antibodies on frozen 5-μm sections of the spleen (**a**–**c**) of mice treated with vehicle (**a**, **c**) or clodronate liposomes (**b**). Quantitative analysis of number of CD68-positive cells per square (=1.5 mm, see **c**) in the liver, spleen and orthotopic MDA-MB-231 tumour of mice treated with vehicle or clodronate liposomes (**d**). Data are mean ± standard deviation (**d**: *n* = 6 (spleen), *n* = 8 (tumour/liver)). Magnification equals ×40 (**a**, **b**) and ×10 (**c**)

Figure 4 shows that macrophage content within the liver (*p* ≤ 0.001), spleen (*p* ≤ 0.01) and tumour (*p* ≤ 0.05) was lowered significantly after treatment with clodronate liposomes, which equalled 363 ± 64, 1,552 ± 274 and 586 ± 260 per square, respectively, when compared to controls (741 ± 216, 2,762 ± 566 and 842 ± 389 per square, respectively). Treatment of mice with empty liposomes did not affect the macrophage content in tissues compared to controls (data not shown).

### MicroSPECT imaging

**Fig. 5:**
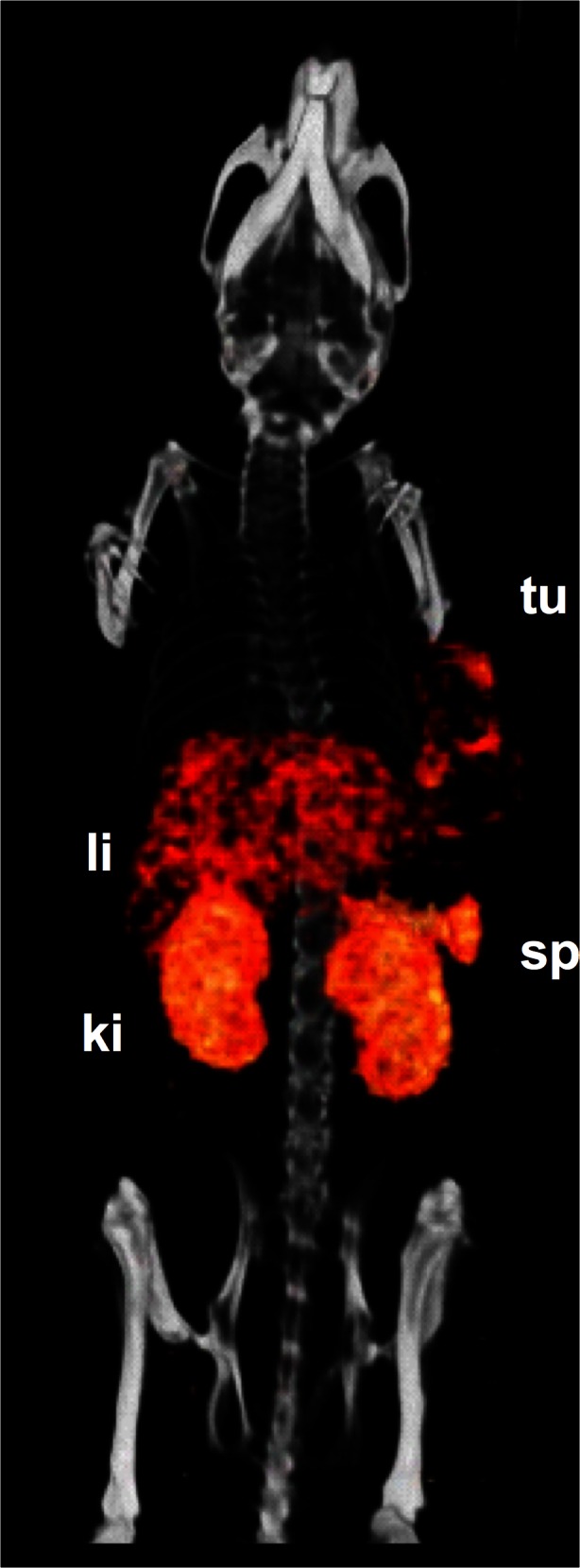
3-D volume projections of SPECT/CT scans taken 24 h p.i. of 100 μg ^111^In-anti-F4/80-A3-1 in mice with orthotopic MDA-MB-231 tumour xenografts. Visible are tumour (*tu*), liver (*li*), spleen (*sp*) and kidneys (*ki*)

Fused SPECT/CT scans (Fig. 5) show that orthotopic MDA-MB-231 tumour xenografts as well as macrophages in the spleen and liver can be visualized. In addition, activity in the kidney was visualized.

## Discussion

In this study, we demonstrated that ^111^In-anti-F4/80-A3-1 allows visualization of TAMs as well as macrophages in the spleen and liver. MicroSPECT imaging also shows uptake of the tracer in the kidneys.

In vitro studies showed that ^111^In-anti-F4/80-A3-1 specifically bound F4/80 receptor-positive macrophages with high affinity. Supplementary Figures 2 and 3 show serum stability up to 24 h after incubation in serum or PBS in vitro. Preliminary ex vivo immunofluorescent staining showed that 21 % of all F4/80-positive cells are targeted by ^111^In-anti-F4/80-A3-1 and that ^111^In-anti-F4/80-A3-1 mostly localized at F4/80-positive cells (77 %; Supplementary Figure 4).

Initial in vivo experiments showed that ^111^In-anti-F4/80-A3-1 at a protein dose of 10 μg per mouse cleared quickly from the blood after injection of the tracer. This was thought to be due to the presence of antigen sinks, mainly in the spleen and liver, where uptake of the tracer was high at 248 and 28 %ID/g at 24 h p.i., respectively. The presence of antigen sinks was confirmed by the observation that liver and spleen uptake were lower when a higher dose of antibody was administered (100 μg per mouse). Partial saturation of these sinks, by the injection of a higher antibody dose, led to improved tumour uptake.

Subsequently, clodronate liposomes were used to decrease the macrophage content within the spleen and liver. These liposomes are selectively ingested by phagocytes and the clodronate, able to kill macrophages, is released within these cells once lysis of the liposomes has occurred [31]. Intravenous application of this liposome-mediated macrophage “suicide” approach decreased the macrophage population within the spleen and liver [31] as well as F4/80-positive macrophages in the tumour microenvironment [32]. Administration of clodronate liposomes decreased uptake of ^111^In-anti-F4/80-A3-1 in the spleen and liver (Fig. 3), which correlated with diminished macrophage density in these organs. Furthermore, treatment of mice with clodronate liposomes decreased the uptake of ^111^In-anti-F4/80-A3-1 in the bone marrow and enhanced blood levels and uptake in the tumour. Despite the tumour to blood ratio for ^111^In-anti-F4/80-A3-1 being similar between control and liposome-treated groups, the specificity of ^111^In-anti-F4/80-A3-1 for macrophages was demonstrated by the higher tumour to blood ratio for ^111^In-anti-F4/80-A3-1 than for ^111^In-rat IgG2b. Treatment with empty liposomes did not affect uptake of ^111^In-anti-F4/80-A3-1 in most tissues and organs (Fig. 3), again indicating that the effects seen after treatment with clodronate liposomes is macrophage-specific, a tentative conclusion also supported by the fact that at injection of 10 μg ^111^In-anti-F4/80-A3-1, 77 % of the tracer localized with the F4/80 receptor (Supplementary Figure 4).

The main exception to this is the liver where treatment with empty liposomes diminished uptake of ^111^In-anti-F4/80-A3-1 compared to the control group. This is thought to be due to the partial saturation in the liver of joint mechanisms involved in opsonization of liposomes as well as of Fc-mediated opsonization of antibodies, here ^111^In-anti-F4/80-A3-1, prior to injection of the tracer [33, 34]. Nevertheless, treatment with clodronate liposomes significantly further diminished ^111^In-anti-F4/80-A3-1 uptake in the liver compared to the group treated with empty liposomes (*p* = 0.01), suggesting that saturated opsonization only partially reduced the tracer uptake and that decreased macrophage density further reduced the hepatic uptake of the tracer.

Macrophage infiltration of the tumour was also affected by clodronate treatment, yet the increase in ^111^In-anti-F4/80-A3-1 uptake in the tumour after clodronate liposomes treatment is a result of enhanced levels of the tracer in the blood.

Apart from fluorescently labelled anti-F4/80-A3-1 antibodies being used to image arthritis in vivo [35], an intact antibody-based approach to visualize tumour-associated macrophages has not previously been attempted. Several targeted approaches have instead included ^99m^Tc-labelled anti-macrophage mannose receptor nanobodies [27] and radiofluorinated folic acid to target the folate receptor on macrophages [28]. Both tracers have a high tumour to blood ratio of approximately 23 and are excellent tracers to follow TAMs for up to 90–180 min p.i. of the tracer. However, with ^111^In-anti-F4/80-A3-1 macrophages could be monitored for up to 72 h p.i. This could be a great advantage when using imaging tracers to monitor response to existing or novel therapies that interfere with macrophage-tumour cell interactions.

Depending on the need of the tracer, the specific kinetics and biodistribution of ^111^In-anti-F4/80-A3-1, or anti-F4/80-A3-1 labelled with a shorter half-life radionuclide to compensate for the shorter than usual circulating time of the antibody, could provide a good alternative to the current armamentarium of macrophage tracers. With an increased interest in developing drug strategies targeting tumour macrophages, a need for imaging these macrophages preclinically has also arisen, which might be met by the tracer presented here, if future studies also prove promising. ^111^In-anti-F4/80-A3-1, as a tracer targeting murine macrophages, therefore has potential to become an important in vivo tool in the development and characterization of novel macrophage-targeted therapies.

In conclusion, ^111^In-anti-F4/80-A3-1 specifically visualized the tumour and macrophages resident in tissues. The spleen and liver act as antigen sink organs, which has implications for the dose of ^111^In-anti-F4/80-A3-1 that should be administered in order to see high tumour targeting. These results imply that if ^111^In-anti-F4/80-A3-1 can be used to monitor TAMs in vivo, its rapid uptake in non-tumour tissues should first be prevented and circulation time enhanced to achieve optimal tumour localization of ^111^In-anti-F4/80-A3-1. Alternatively, imaging TAMs could also be further accomplished by developing a tracer specific to M2 macrophages. This should lead to a comparatively enhanced tumour uptake and diminished non-tumour tissue uptake by circumventing M1 macrophages that were present in the current antigen sinks leading to reduced tumour uptake of ^111^In-anti-F4/80-A3-1.

## Electronic supplementary material

Supplementary Figure 1 (DOCX 35 kb)

Supplementary Figure 2 (DOCX 1740 kb)

Supplementary Figure 3 (DOCX 104 kb)

Supplementary Figure 4 (DOCX 170 kb)
