# Supplementary material for: 111In-anti-F4/80-A3-1 antibody: a novel tracer to image macrophages
Source: Eur J Nucl Med Mol Imaging. 2015 May 27;42(9):1430–8. doi: 10.1007/s00259-015-3084-8 (PMC4502320; doi:10.1007/s00259-015-3084-8)
Supplement: Supplementary file 1 — (DOCX 35 kb) [file 259_2015_3084_MOESM1_ESM.docx]

Supplementary Figure 1

Binding plot and Lindmo plot of ^111^In-anti-F4/80-A3-1. 1/intercept:= immunoreactive fraction x100% = 75%.
