# Supplementary material for: 111In-anti-F4/80-A3-1 antibody: a novel tracer to image macrophages
Source: Eur J Nucl Med Mol Imaging. 2015 May 27;42(9):1430–8. doi: 10.1007/s00259-015-3084-8 (PMC4502320; doi:10.1007/s00259-015-3084-8)

Supplementary Figure 2

Labeling efficiency and radiochemical purity were determined using instant thin-layer chromatography on silica gel strips (Varian-Agilent; Agilent Technologies Netherlands B.V.; Middelburg, the Netherlands) with 0.15 M sodium citrate buffer, pH 5.5, as the mobile phase.

ITLC of 111In-anti-F4/80-A3-1 before and after 1:1: (v:v) incubation in PBS or serum for 2 and 24 hours.

111In-anti-F4/80-A3-1


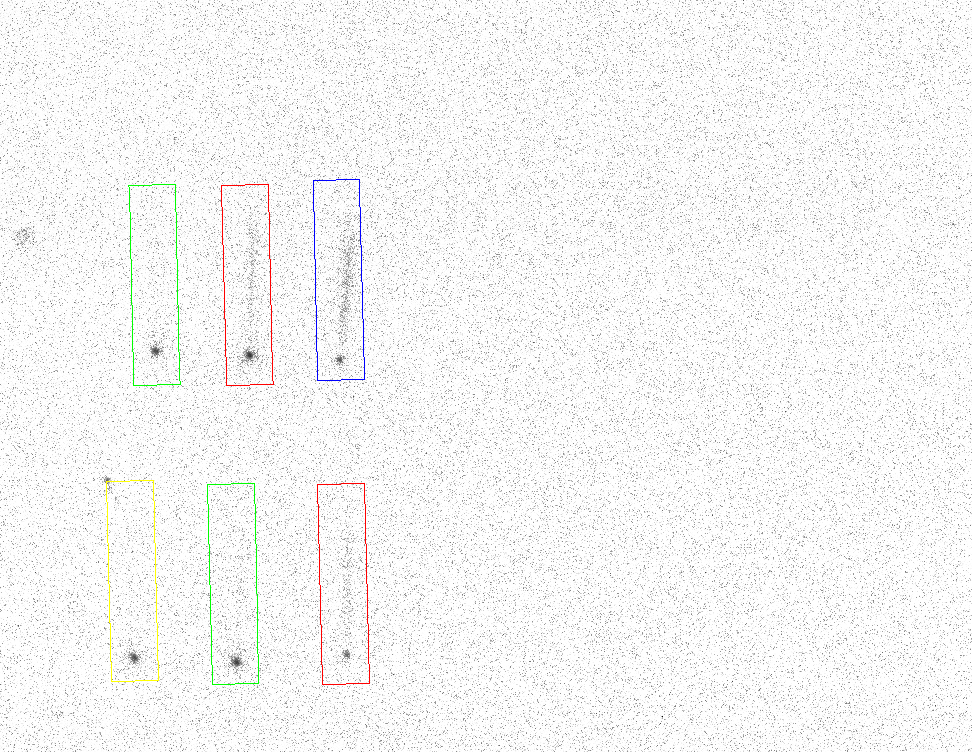
0hr

2hr 24hr


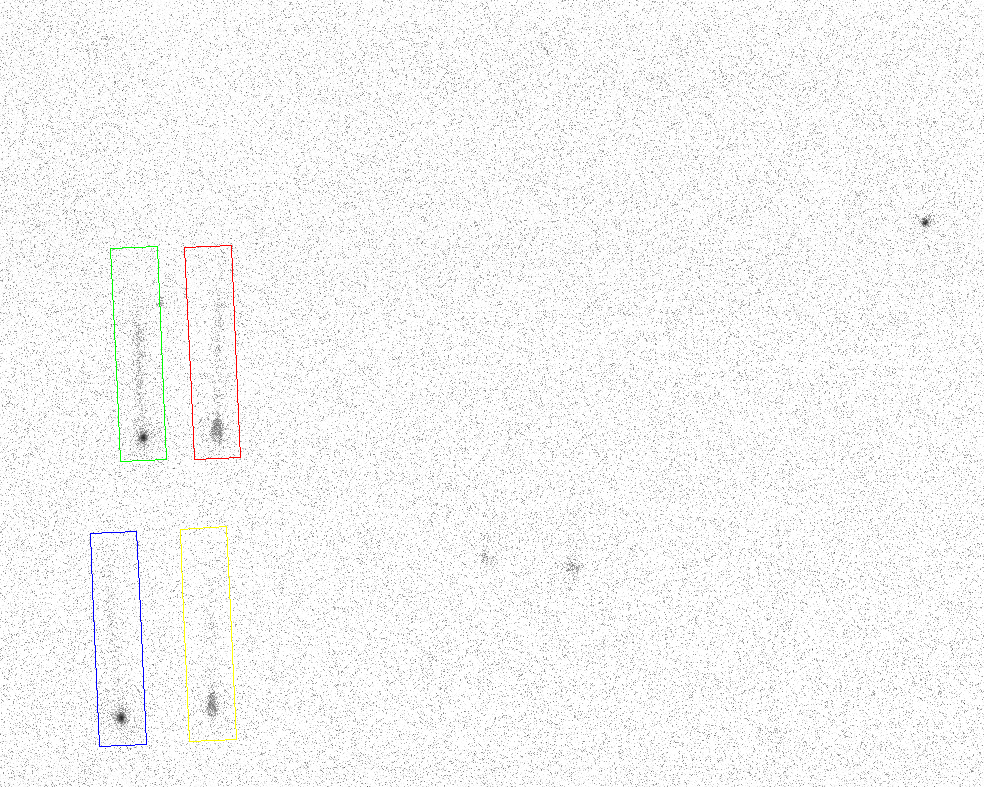


F4/80-A3-1

PBS Serum

All 100%

PBS Serum

F4/80-A3-1

All 100%


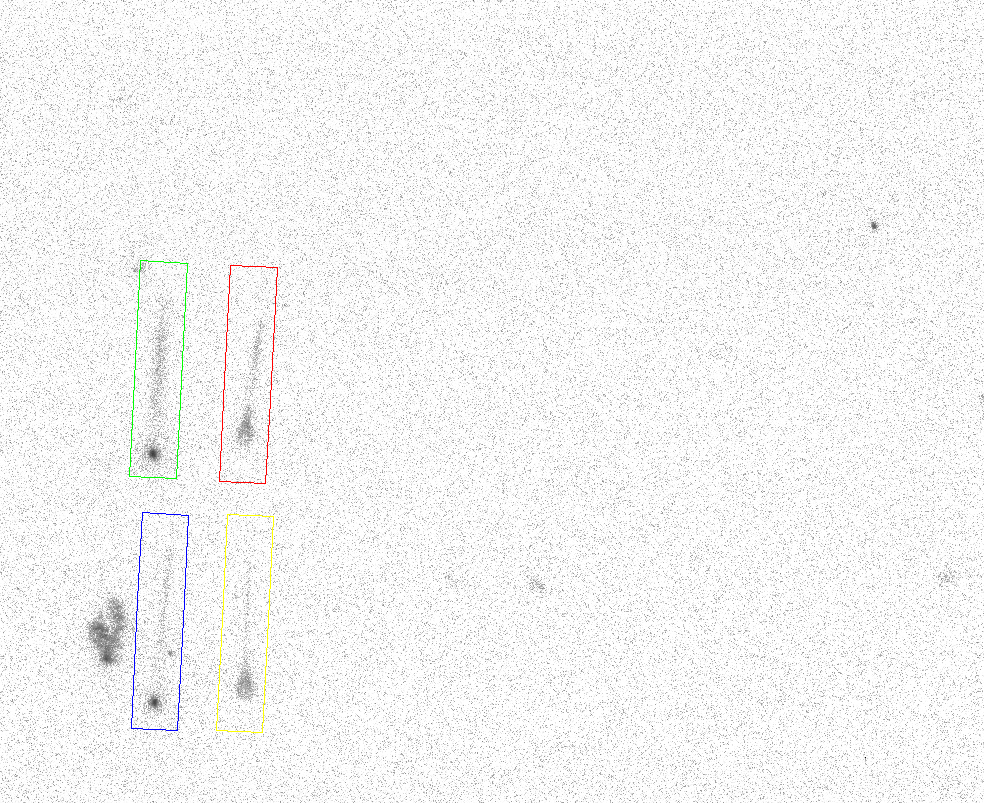

Supplement: Supplementary file 2 — (DOCX 1740 kb) [file 259_2015_3084_MOESM2_ESM.docx]
