# Supplementary material for: 111In-anti-F4/80-A3-1 antibody: a novel tracer to image macrophages
Source: Eur J Nucl Med Mol Imaging. 2015 May 27;42(9):1430–8. doi: 10.1007/s00259-015-3084-8 (PMC4502320; doi:10.1007/s00259-015-3084-8)
Supplement: Supplementary file 3 — (DOCX 104 kb) [file 259_2015_3084_MOESM3_ESM.docx]

Supplementary Figure 3

Quality control was also performed using fast protein liquid chromatography (FPLC) on a BioSep-Sec-S3000 column (300 x 7.80 mm; Phenomenex) eluted with 1 mL/min PBS. Preparations were analyzed on an Agilent 1200 system (Agilent Technologies, Palo Alto, CA, USA). Radioactivity was monitored using an in-line NaI radiodetector (Raytest GmbH, Straubenhardt, Germany) and elution profiles were analyzed using Gina-star software (Raytest GmbH).

FPLC (UV: A,B,E and Radiochromatogram: C-E) of (111In-)anti-F4/80-A3-1. UV traces are of anti-F4/80-A3-1 pre and post dialysis of ITC-DTPA (A-B). Radiochromatograms are of 111In-anti-F4/80-A3-1 before and after incubation in serum (1:1 v:v) up to 48 hours.

A

Pre-dialysis (100ug) – UV

🡪 ITC-DTPA-F4/80-A3-1 – 18min

🡪 excess of ITC-DTPA: 23min

B

Post-dialysis (100ug ) – UV

🡪 ITC-DTPA-F4/80-A3-1 –18min

🡪 No excess ITC-DTPA at 23min

C

Radiochromatogram

111In-anti-F4/80-A3-1 0hr

D

Radiochromatogram

111In-anti-F4/80-A3-1 24hr in serum

E – UV (smooth) and Radiochromatograms (jagged)

0h

1h

4h

24h

48h
