# Supplementary material for: 111In-anti-F4/80-A3-1 antibody: a novel tracer to image macrophages
Source: Eur J Nucl Med Mol Imaging. 2015 May 27;42(9):1430–8. doi: 10.1007/s00259-015-3084-8 (PMC4502320; doi:10.1007/s00259-015-3084-8)
Supplement: Supplementary file 4 — (DOCX 170 kb) [file 259_2015_3084_MOESM4_ESM.docx]

Supplementary Figure 4

Ex vivo immunofluorescence staining of 10um frozen sections of MDA-MB-231 tumors in a untreated mouse, injected with 10ug 111In-anti-F4/80-A3-1. Colocalization studies of anti-F4/80-PE (staining for F4/80 receptor at different epitope than 111In-F4/80-A3-1) and anti-ratIgG2b-FITC (staining for injected tracer).

F4/80-A3-1-PE, RatIgG2b-FITC and Hoechst (blue) – tumor untreated mouse X400 magn.


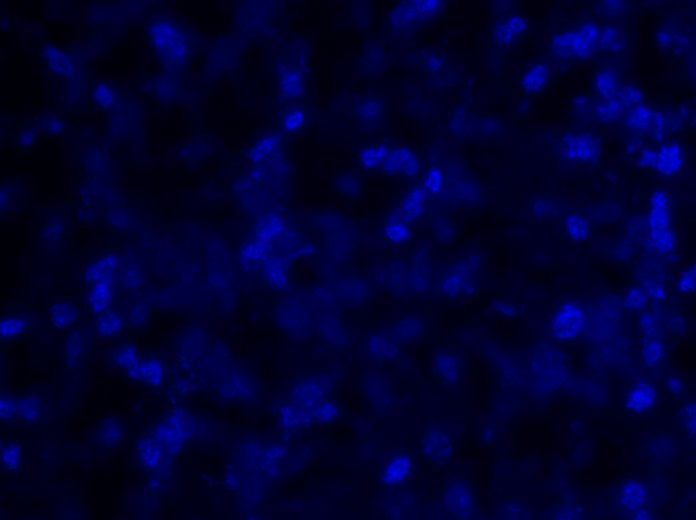

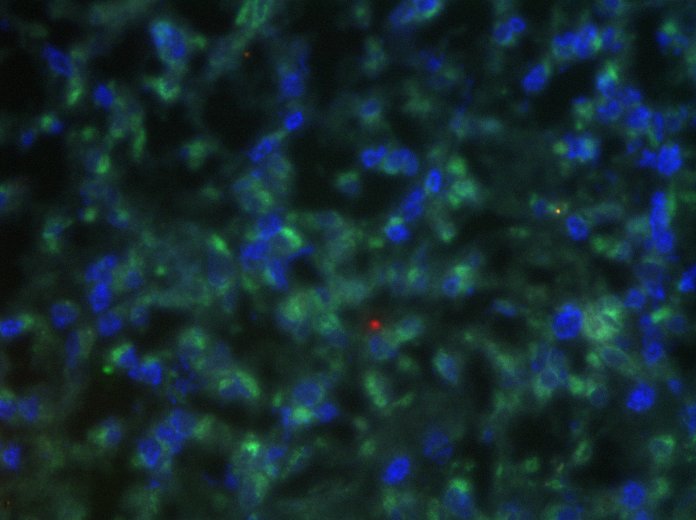

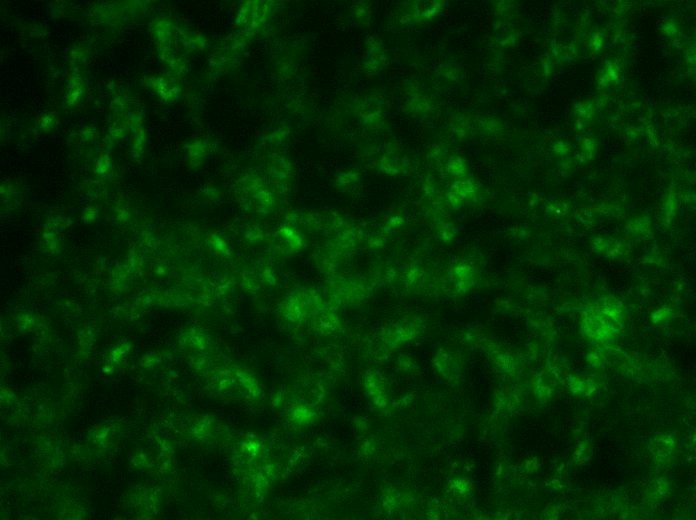

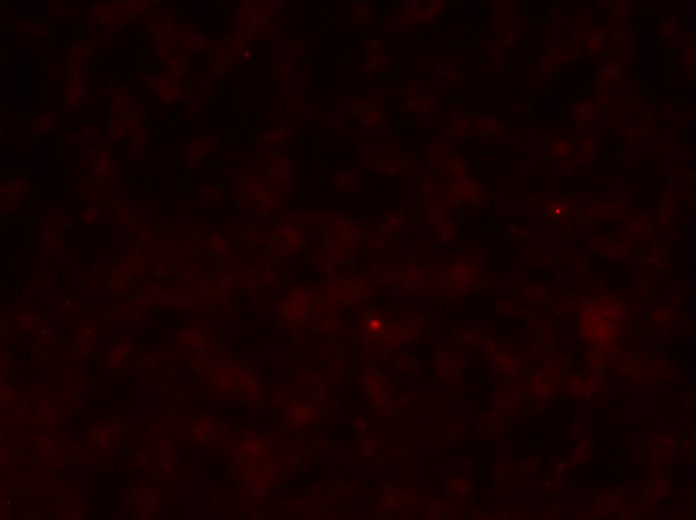


|  | Percentage | |
| --- | --- | --- |
|  | F4/80-A3-1 that is  ratIG2b+ | ratIgG2b that is F4/80+ |
| Tumor | 21 | 77 |
|  |  |  |
